# Supplementary material for: Content-rich biological network constructed by mining PubMed abstracts
Source: BMC Bioinformatics. 2004 Oct 8;5:147. doi: 10.1186/1471-2105-5-147 (PMC528731; doi:10.1186/1471-2105-5-147)
Supplement: Additional File 2 — The original results of the above study (non-essential files are deleted to keep the file size under the limit set by BMC bioinformatics). [file 1471-2105-5-147-S2.bz2 › chilibotAdditionalFile2/dip40/746ID7744737E18126/html/TOLA_TOLQ.html]

 


 **TOLA** and **TOLQ** 
  
Found 36 abstracts in PubMed, retrieved 36.  
 

 What does Google say? 
 PDF only 
| .edu only 

---

**Interactive relationship** (e.g. stimulation, inhibition, etc)

**Neutral relationship**- Role of TolR N terminal, central, and C terminal domains in dimerization and interaction with  **TolA**  and  **tolQ** .  Ref: 10419942 J Bacteriol, 1999
- The  **TolQ**  TolR proteins energize  **TolA**  and share homologies with the flagellar motor proteins MotA MotB.  Ref: 11722743 Mol Microbiol, 2001
- Cross linking experiments have shown that the  **TolA**  transmembrane domain interacts with  **TolQ**  and TolR.  Ref: 10419942 J Bacteriol, 1999
- **TolA**  N terminal domain interacts with  **TolQ**  and TolR proteins.  Ref: 7744737 J Biol Chem, 1995
- These data demonstrate that  **TolA**  interacts directly with TolR and  **TolQ** .  Ref: 7744737 J Biol Chem, 1995
- These results confirmed the essential role of the transmembrane domain of  **TolA**  in triggering interactions with  **TolQ**  and TolR.  Ref: 9851983 J Bacteriol, 1998
- A point mutation within the third transmembrane TM segment of  **TolQ**  was found to affect the  **TolA**  Pal interaction strongly.  Ref: 11722743 Mol Microbiol, 2001
- Moreover, immunoprecipitation experiments with the antiserum directed against  **TolA**  indicated that  **TolQ**  and TolR were co immunoprecipitated with  **TolA**  after cross linking.  Ref: 7744737 J Biol Chem, 1995
- The N terminal transmembrane domain of  **TolA**  anchors the protein to the cytoplasmic membrane and interacts with  **TolQ**  and TolR.  Ref: 9851983 J Bacteriol, 1998
- The TolR transmembrane domain was shown to be involved in the  **TolA**  TolR and  **TolQ**  TolR interactions, while TolR central and C terminal domains appeared to be involved in TolR dimerization.  Ref: 10419942 J Bacteriol, 1999
- Energy dependent conformational change in the  **TolA**  protein of Escherichia coli involves its N terminal domain,  **TolQ** , and TolR.  Ref: 11418549 J Bacteriol, 2001
- Therefore, the reduction in O7 LPS was attributed to the lack of a functional  **tolA**  gene, caused either by a direct mutation of this gene or by a polar effect on  **tolA**  gene expression exerted by the  **tolQ**  mutation.  Ref: 11069653 Mol Microbiol, 2000

**Non-interactive relationship** (e.g. studied together, co-existance, homology, etc.)

- Bacteriocin sensitivity tests performed on a collection of E. coli envelope mutants, and isolation and characterization of E. coli bacteriocin 28b insensitive mutants, showed that the core lipopolysaccharide, outer membrane proteins OmpA and OmpF, and  **TolQ** ,  **TolA** , and TolB proteins are involved in bacteriocin 28b lethal activity.  Ref: 8595593 Can J Microbiol, 1996
- The  **TolA** , TolB,  **TolQ** , and TolR proteins are involved in maintaining the integrity of the Escherichia coli outer membrane and in the import of group A colicins and filamentous phage DNA.  Ref: 7744737 J Biol Chem, 1995
- The O7 deficient phenotype in the  **tolQ**  and  **tolA**  mutants was complemented with a plasmid encoding the tolQRA operon, but not with a similar plasmid containing a frameshift mutation inactivating  **tolA** .  Ref: 11069653 Mol Microbiol, 2000
- Analysis of plasmid encoded  **tolQ**  lacZ and  **tolA**  lacZ promoter fusions in E. coli indicated that these genes are expressed at different levels, suggesting transcription from different promoters.  Ref: 8955385 J Bacteriol, 1996
- The nucleotide sequence of a 2.9 kb DNA fragment containing the  **tolQ** , tolR, and  **tolA**  genes was determined.  Ref: 8955385 J Bacteriol, 1996
- **TolQ** , TolR, and  **TolA**  are located in the cytoplasmic membrane where they interact with each other via their transmembrane domains.  Ref: 10633120 J Bacteriol, 2000
- However, strains with mutations in both tcpA and either  **tolQ** , tolR, or  **tolA**  were completely resistant to CTXphi infection.  Ref: 10692381 J Bacteriol, 2000
- The  **tolA**  and  **tolQ**  mutants showed reduced O7 LPS expression compared with the respective isogenic parent strains.  Ref: 11069653 Mol Microbiol, 2000
- This cluster was found to consist of seven genes in the order orf1  **tolQ**  tolR  **tolA**  tolB oprL orf2.  Ref: 10735848 J Bacteriol, 2000
- Suppressor mutant analyses have localized the  **TolQ**   **TolA**  interaction to the first transmembrane domain of  **TolQ**  and have shown that the third transmembrane domain of  **TolQ**  interacts with the transmembrane domain of TolR.  Ref: 10419942 J Bacteriol, 1999
- Mutations affecting the function of  **TolA**  resulted in a lack or an alteration in  **TolA**   **TolQ**  or TolR  **TolA**  interactions but did not affect the formation of  **TolQ**  TolR complexes.  Ref: 9851983 J Bacteriol, 1998
- This gene is located between fii and  **tolA** , and we propose the designation of tolQRA for this cluster in which  **tolQ**  is the former fii gene and tolR is the new open reading frame.  Ref: 3294803 J Bacteriol, 1987
- Mutations in  **tolQ** , tolR,  **tolA** , tolB, or pal genes result in sensitivity to bile salts and the leakage of periplasmic proteins.  Ref: 10633120 J Bacteriol, 2000
- In this study, we show that both  **TolQ**  and TolR proteins are essential for the  **TolA**  Pal interaction.  Ref: 11722743 Mol Microbiol, 2001
- 5 fold decrease in cytotoxicity of the  **TolA**  dependent colicins A, E1, E3, and N compared to cells producing wild type  **TolA**  when these mutants were expressed alone or with  **TolQ** , R, and B.  Ref: 9171417 J Bacteriol, 1997
- In the present study we cloned and mutagenized the orf1,  **tolQ** , tolR,  **tolA** , and tolB genes from P. putida KT2440, which were located upstream of the oprL gene.  Ref: 10940016 J Bacteriol, 2000
- Activity of ExbB ExbD via  **TolA**  was higher in cells lacking TonB, and activity of  **TolQ**  TolR via TonB was increased when  **TolA**  was missing.  Ref: 8316079 Mol Microbiol, 1993
- The  **tolQ** , tolR, and  **tolA**  genes from Pseudomonas aeruginosa PAO were cloned using degenerate oligonucleotide PCR primers designed based on conserved transmembrane regions of Escherichia coli  **TolQ**  and TolR and E. coli and Pseudomonas putida ExbB and ExbD.  Ref: 8955385 J Bacteriol, 1996
- This infection is dependent on an intact particle and requires the products of the  **tolQ** , tolR, and  **tolA**  genes of the bacteria.  Ref: 3053655 J Bacteriol, 1988
- Group B colicins, ferric siderophores and vitamin B12 are taken up via the TonB ExbB ExbD, group A colicins via the  **TolA**   **TolQ**  TolR system.  Ref: 8316079 Mol Microbiol, 1993
- The deduced amino acid aa sequence of the H. influenzae  **TolQ** , TolR,  **TolA**  and TolB show 6 1 and 62% identity with Escherichia coli TolQRAB proteins, respectively.  Ref: 8921895 Gene, 1996
- Transmembrane domains of  **TolQ** , TolR and  **TolA**  interact in the cytoplasmic membrane, while TolB and Pal form a complex near the outer membrane.  Ref: 9701827 Mol Microbiol, 1998
- It is organized into two complexes, one near the outer membrane between TolB and PAL and one in the cytoplasmic membrane between  **TolA** ,  **TolQ** , and TolR.  Ref: 10419942 J Bacteriol, 1999
- In this study, we demonstrate that the genes  **tolQ** , tolR, and  **tolA** , but not tolB, tolC, and ompF, are required for the internalization of cloacin DF13 and they are not involved in the transport of ferric aerobactin.  Ref: 8419302 J Bacteriol, 1993
- Uptake of the mutated colicin U DTMVV depended on TonB and ExbB and still remained  **TolA**  and  **TolQ**  dependent, but no longer required TolB.  Ref: 9682486 FEMS Microbiol Lett, 1998
- **TolA** ,  **TolQ** , and TolR are inner membrane proteins, TolB is a periplasmic protein, and Pal, the peptidoglycan associated lipoprotein, is anchored to the outer membrane.  Ref: 11790745 J Bacteriol, 2002
- **TolA** ,  **TolQ** , and TolR are localized in the inner membrane while TolB is periplasmic, although a small amount of membrane associated TolB is always found.  Ref: 7744737 J Biol Chem, 1995
- Evolutionary relationship of uptake systems for biopolymers in Escherichia coli cross complementation between the TonB ExbB ExbD and the  **TolA**   **TolQ**  TolR proteins.  Ref: 8316079 Mol Microbiol, 1993
- Entry of the hybrid toxin was also shown to depend on the products of  **tolA** ,  **tolQ** , and tolR which are required both for phage f1 infection and for entry of E colicins.  Ref: 3045089 J Bacteriol, 1988
- This change requires  **TolQ** , TolR, and a functional  **TolA**  N terminal domain.  Ref: 11418549 J Bacteriol, 2001
- Group A colicins need proteins of the Escherichia coli envelope Tol complex  **TolA** , TolB,  **TolQ**  and TolR to reach their cellular target.  Ref: 9466263 Mol Microbiol, 1998
- Analysis of polar mutations, reverse transcriptase PCR assays, and transcriptional fusion constructs with a promoterless lacZ gene revealed that the genes are arranged in two operons orf1  **tolQ**  tolR  **tolA**  tolB and oprL orf2.  Ref: 12486055 J Bacteriol, 2003
- Bacteria carrying  **tolA** ,  **tolQ**  and tolR mutations kept their colicin insensitivity as spheroplasts, just as the resistant ones.  Ref: 12094727 Folia Microbiol Praha , 2002
- Since these two  **TolA**  derivatives still formed specific complexes with TolR derivatives still formed specific complexes with TolR and  **TolQ** , we concluded that the  **TolA**  N terminal domain interacted with these proteins.  Ref: 7744737 J Biol Chem, 1995
- The Tol Pal system of the Escherichia coli envelope is formed from the inner membrane  **TolQ** , TolR and  **TolA**  proteins, the periplasmic TolB protein and the outer membrane Pal lipoprotein.  Ref: 11115123 Mol Microbiol, 2000
- The interactions of TolR with  **TolA**  and  **TolQ**  were confirmed, TolR was shown to dimerize, and the resulting dimer was shown to interact with  **TolQ** .  Ref: 10419942 J Bacteriol, 1999
- They first interact with receptors located at the surface of the outer membrane and are then transferred across the cell envelope in a process that requires energy and depends upon accessory proteins  **TolA** , TolB, TolC,  **TolQ** , TolR which might play a role similar to that of the secretory apparatus of eukaryotic and prokaryotic cells.  Ref: 3139035 Biochim Biophys Acta, 1988
- It was shown that the  **TolA** ,  **TolQ** , and TolR proteins play a critical role in the translocation of group A colicins and filamentous bacteriophage minor coat proteins g3p.  Ref: 11851406 Biochemistry, 2002
- Polar and nonpolar mutations of the P. putida  **tolQ** , tolR,  **tolA** , and tolB genes were generated in vitro by using the omega Km r interposon, which carries two transcriptional stop signals, or a promoterless xylE cassette, lacking any transcriptional stop signal, respectively.  Ref: 10940016 J Bacteriol, 2000
- In contrast, a polar  **tolQ**  mutation affecting  **tolA**  expression in VW187 caused slow growth rate and serum sensitivity in addition to reduced O7 LPS production.  Ref: 11069653 Mol Microbiol, 2000
- The  **TolQ** , TolR,  **TolA** , TolB, and Pal proteins appear to function in maintaining the integrity of the outer membrane, as well as facilitating the uptake of the group A colicins and the DNA of the infecting filamentous bacteriophages.  Ref: 8763928 J Bacteriol, 1996
- Attempts to inactivate the chromosomal copy of the  **tolA**  or  **tolQ**  gene in the parent strain PAO proved to be unsuccessful, and we propose that inactivation of these genes in P. aeruginosa results in a lethal phenotype.  Ref: 8955385 J Bacteriol, 1996
- The Tol Pal system of Escherichia coli is composed of the YbgC,  **TolQ** ,  **TolA** , TolR, TolB, Pal and YbgF proteins.  Ref: 11994151 Mol Microbiol, 2002
- In Pseudomonas putida, the seven genes, orf1,  **tolQ** , tolR,  **tolA** , tolB, oprL, and orf2, which encode the proteins of this complex, are clustered in a 5.8 kb region of chromosomal DNA.  Ref: 12486055 J Bacteriol, 2003
- The  **TolQ**  TolR  **TolA**  proteins form a complex in the cytoplasmic membrane, while TolB and Pal interact near the outer membrane.  Ref: 10474183 FEMS Microbiol Lett, 1999
- **TolA**  and TolR are anchored to the inner membrane by a single transmembrane domain,  **TolQ**  is an integral membrane protein with three transmembrane segments, and TolB has recently been found to be periplasmic although it is partially membrane associated.  Ref: 7744736 J Biol Chem, 1995
- **TolQ** , TolR and  **TolA**  are membrane proteins involved in maintaining the structure of Escherichia coli cell envelope.  Ref: 7853390 J Mol Biol, 1995
- Mutational analysis of the Escherichia coli K 12  **TolA**  N terminal region and characterization of its  **TolQ**  interacting domain by genetic suppression.  Ref: 9851983 J Bacteriol, 1998
- Transmembrane domains of  **TolQ** , TolR, and  **TolA**  interact in the cytoplasmic membrane, while TolB and Pal form a complex near the outer membrane.  Ref: 9851983 J Bacteriol, 1998
- A promoterless lacZ gene was introduced downstream of plasmid encoded  **tolQ**  and  **tolA** , and expression was monitored by measuring beta galactosidase activity of cultures.  Ref: 9603869 J Bacteriol, 1998
- The resulting PCR product was used as a probe to isolate a 6.5 kb DNA fragment containing P. aeruginosa  **tolQ** , tolR, and  **tolA** .  Ref: 8955385 J Bacteriol, 1996
- Isolation and characterization of the Haemophilus influenzae  **tolQ** , tolR,  **tolA**  and tolB genes.  Ref: 8921895 Gene, 1996
- Subsequent translocation of phage DNA requires the chromosomally encoded  **TolQ** , TolR, and  **TolA**  proteins, after the pilus presumably has withdrawn, bringing the phage to the bacterial surface.  Ref: 9335297 J Bacteriol, 1997
- V. cholerae strains with insertion mutations in  **tolQ** , tolR, or  **tolA**  were reduced in their efficiency of CTXphi uptake by 4 orders of magnitude.  Ref: 10692381 J Bacteriol, 2000
- Furthermore, bacteriocin 28b translocation requires proteins  **TolA** , TolB, and  **TolQ** .  Ref: 8595593 Can J Microbiol, 1996
- Sequence data showed that these genes are clustered in a 6 kb segment of DNA with the gene order orf1  **tolQ**  tolR  **tolA**  tolB pal orf2 a newly identified open reading frame encoding a 29 kD9 protein.  Ref: 8763928 J Bacteriol, 1996
- DNA transfer requires the chromosomally encoded  **TolA** ,  **TolQ** , and TolR cytoplasmic membrane proteins.  Ref: 9537368 J Bacteriol, 1998
- **TolQ** , TolR, and  **TolA**  inner membrane proteins of Escherichia coli are involved in maintaining the stability of the outer membrane.  Ref: 11418549 J Bacteriol, 2001
- **TolQ**  TolR interacted through TonB, and ExbB ExbD interacted through  **TolA**  with the outer membrane receptors and colicins.  Ref: 8316079 Mol Microbiol, 1993
- The predicted amino acid sequences of V. cholerae  **TolQ** , TolR,  **TolA** , and TolB showed significant similarity to the corresponding E. coli sequences.  Ref: 10692381 J Bacteriol, 2000
- This fragment was also detected in Delta  **tolQ** , Delta tolR, and  **tolA**  H22P mutants but, in contrast to the parental strain, was also seen in the absence of uncouplers.  Ref: 11418549 J Bacteriol, 2001
